# Colonisation resistance in the sand fly gut: *Leishmania* protects *Lutzomyia longipalpis* from bacterial infection

**DOI:** 10.1186/1756-3305-7-329

**Published:** 2014-07-23

**Authors:** Mauricio RV Sant’Anna, Hector Diaz-Albiter, Kelsilândia Aguiar-Martins, Waleed S Al Salem, Reginaldo R Cavalcante, Viv M Dillon, Paul A Bates, Fernando A Genta, Rod J Dillon

**Affiliations:** Faculty of Health and Medicine, Division of Biomedical and Life Sciences, Lancaster University, Lancaster, UK; Departamento de Parasitologia, ICB/UFMG, Belo Horizonte, Brazil; Instituto Oswaldo Cruz, Fundação Oswaldo Cruz, Rio de Janeiro, Brazil; Departamento de Parasitologia, ICB/UFMG, Belo Horizonte, Brazil; Liverpool School of Tropical Medicine, Vector Group, Liverpool, UK; Parasitologia e Microbiologia, CCS, Universidade Federal do Piauí, Teresina, Piauí Brazil; Institute of Integrative Biology, University of Liverpool, Liverpool, UK

**Keywords:** *Leishmania*, *Lutzomyia*, *Asaia*, *Pseudozyma*, *Serratia*, Sand fly, Microbiota

## Abstract

**Background:**

Phlebotomine sand flies transmit the haemoflagellate *Leishmania*, the causative agent of human leishmaniasis. The *Leishmania* promastigotes are confined to the gut lumen and are exposed to the gut microbiota within female sand flies. Here we study the colonisation resistance of yeast and bacteria in preventing the establishment of a *Leishmania* population in sand flies and the ability of *Leishmania* to provide colonisation resistance towards the insect bacterial pathogen *Serratia marcescens* that is also pathogenic towards *Leishmania*.

**Methods:**

We isolated microorganisms from wild-caught and laboratory-reared female *Lutzomyia longipalpis*, identified as *Pseudozyma* sp. *Asaia* sp. and *Ochrobactrum intermedium*. We fed the females with a sugar meal containing the microorganisms and then subsequently fed them with a bloodmeal containing *Leishmania mexicana* and recorded the development of the *Leishmania* population. Further experiments examined the effect of first colonising the sand fly gut with *L. mexicana* followed by feeding with, *Serratia marcescens,* an insect bacterial pathogen. The mortality of the flies due to *S. marcescens* was recorded in the presence and absence of *Leishmania*.

**Results:**

There was a reduction in the number of flies harbouring a *Leishmania* population that had been pre-fed with *Pseudozyma* sp. and *Asaia* sp. or *O. intermedium*. Experiments in which *L. mexicana* colonised the sand fly gut prior to being fed an insect bacterial pathogen, *Serratia marcescens,* showed that the survival of flies with a *Leishmania* infection was significantly higher compared to flies without *Leishmania* infection.

**Conclusions:**

The yeast and bacterial colonisation experiments show that the presence of sand fly gut microorganisms reduce the potential for *Leishmania* to establish within the sand fly vector. Sand flies infected with *Leishmania* were able to survive an attack by the bacterial pathogen that would have killed the insect and we concluded that *Leishmania* may benefit its insect host whilst increasing the potential to establish itself in the sand fly vector. We suggest that the increased ability of the sand fly to withstand a bacterial entomopathogen, due to the presence of the *Leishmania*, may provide an evolutionary pressure for the maintenance of the *Leishmania*-vector association.

**Electronic supplementary material:**

The online version of this article (doi:10.1186/1756-3305-7-329) contains supplementary material, which is available to authorized users.

## Background

Female phlebotomine sand flies are the primary vectors for transmission of the medically important haemoflagellate *Leishmania* between their mammalian hosts [1, 2]. *Leishmania* develop exclusively within the gut of the female insect [3] and this means that *Leishmania* will be exposed to other microorganisms that are either resident or passing through the gut. Adult sand flies acquire their microbiota from several sources: during blood feeding on their animal hosts; from plants on which the adults feed and via the pupal stage from exposure of larval stages to their terrestrial dwelling sites [4–8].

The concept of colonisation resistance is well established in vertebrate gut systems [9]. This is the ability of the gut microbiota to prevent the development of invasive microorganisms, commonly potential pathogens, by a combination of direct microbial interaction and indirect actions of the mucosal immune system. A similar concept has been presented for insects [10]. A role for insect gut bacteria in colonisation resistance towards medically important parasites has been established in other insects [10–14]. In these studies, the gut microbiota may reduce transmission of the medically important parasite rather than prevent development of an insect pathogen. Nearly 30 years ago, Schlein *et al.* discussed the possibility of gut microorganisms in Phlebotomine sand flies preventing *Leishmania* development [15]. In the first part of our study, we explored the effects of yeast and bacterial colonisation of the female sand fly gut on the subsequent establishment of *Leishmania* in the gut of the vector. The microorganisms used for the investigation were the bacteria *Asaia* sp., *Ochrobactrum intermedium* and a yeast-like fungus *Pseudozyma* sp. from the gut of field-caught or lab-reared *Lu. longipalpis*.

In the second part of our study, we addressed the idea that the *Leishmania* might promote colonisation resistance for the sand fly host. Adult sand flies are short lived, averaging perhaps 10 days in the wild [16]. For successful transmission during that time the sand fly must first feed on a *Leishmania*-infected mammal to acquire the parasites, these must then colonise the gut, and the insect must remain healthy for long enough to transfer *Leishmania* to another mammal when the *Leishmania* is regurgitated during the next blood feed [17]. The benefit for *Leishmania* in its relationship with the insect is obvious, but there are fitness costs for the sand fly: *Leishmania* populations in the gut can lead to a reduction in *Lu. longipalpis* longevity and this is effect is enhanced if infected flies are stressed [3, 18]. It is known that *Leishmania* can modulate the digestive enzymes of the sand fly host [19–21] and may cause damage to the stomodeal valve in the gut [22]. There is also evidence for an insect immune response towards *Leishmania* and if the sand fly immune system is appropriately stimulated this can lead to a loss of *Leishmania* infection [23]. However, the idea that a *Leishmania* infection may be of advantage to the sand fly has never been explored; even intermittent benefits of *Leishmania* towards its vector will have a profound influence on the relationship. In the second part of the study we designed experiments to explore the potential benefits to the sand fly host in harbouring *Leishmania* in the gut. We tested the hypothesis to see if the presence of *Leishmania* could provide colonisation resistance towards entomopathogenic bacteria. These experiments were performed using the sand fly *Lu. longipalpis* [24] together with *Leishmania mexicana,* an excellent model to study *Leishmania-*sand fly interactions [17], and *Serratia marcescens* an insect bacterial pathogen. This bacterium was previously found associated with wild *Lu. longipalpis* [7] and is also lethal to *Leishmania in vitro* [25].

## Methods

### Ethics statement

All procedures involving animals were approved by the ethical review committee of the University of Liverpool and performed in accordance with United Kingdom Government (Home Office approved project licence PPL40/2958) and EC regulations.

### Sand fly rearing

All experiments were performed using *Lu. longipalpis* obtained originally from Jacobina (Bahia-Brazil) and reared under standardised laboratory conditions [26]. Adult insects were kept under controlled temperature (27 ± 2°C), humidity (> 80%) and photoperiod (8 hours light/16 hours darkness) and were fed on a diet consisting of autoclaved 70% w/v sucrose solution on cotton wool *ad libitum*. Female sand flies were blood fed once a week with EDTA-treated rabbit blood via a Hemotek membrane feeder (Discovery Workshops-UK) at 37°C.

### Isolation of microorganisms from sand flies

Wild-caught *Lu. longipalpis* were captured in Teresina, an endemic area under active urban transmission of visceral leishmaniasis in Northeast Brazil. The midgut from each single surface sterilised fly (70% v/v ethanol for 3 mins) was homogenised in 50 μl of PBS and serial dilutions were inoculated onto LB/agar plates and incubated at 26°C for 24-72 h. Selected colonies of field-isolated bacteria and yeast-like fungi were identified by PCR amplification using primers for the 16 or 26S rRNA gene. Full-length sequences of the 16S rRNA gene were obtained for field-isolated bacteria *Asaia* sp. (HE995765) and *Ochrobactrum intermedium* (HE995764) isolated from our laboratory colony *Lu. longipalpis* using previously described primers [27]. A yeast-like fungus *Pseudozyma* sp (KJ493325) was identified from a wild caught sand fly using primers NL-1 and NL-4, designed for yeasts, for the variable D1/D2 domain of the large subunit (26S) ribosomal DNA sequence [28].

### Sand fly infections

Sand fly infections with promastigotes of *L. mexicana* (strain MNYC/BZ/62/M379) were performed as described previously [29]. Heat-inactivated (56°C for 1 hour) rabbit blood was used to re-suspend cultured promastigotes to a final concentration of 1 × 10^6^ parasites mL^-1^ in comparison to a control without heat inactivation. Rabbit blood seeded with parasites was offered to *Lu. longipalpis* 96 hours after bacterial or yeast feed. Infected sand flies were then transferred to new cages, dissected 72 hours after infection and the number of *Leishmania* promastigotes inside their midguts was recorded using a haemocytometer.

### Colonisation resistance experiments

*Pseudozyma* (strain Pa1), *Asaia* sp. (A1) and *Ochrobactrum intermedium* (Om17) were inoculated on GLY/agar plates (contains nutrients 2.5% w/v glycerol and 1% w/v yeast extract) or Luria-Bertani agar (*O. intermedium*) and incubated overnight for 24 hours at 26°C. Single colonies were transferred to media in 50 mL polypropylene tubes and grown in a shaking incubator (200 rpm). Growth conditions were; 18 h in GLY media (pH = 5.5) at 30°C (*Pseudozyma* sp.) and 72 h (*Asaia* sp.); 18 h in 40 mL of LB media at 37°C (*O. intermedium*). The microbial suspensions were centrifuged at 6,500 g for 5 minutes and re-suspended in 0.22 μm-filtered 7% sucrose solution to a concentration between 2- 4 × 10^7^ CFU mL^-1^. When *Pseudozyma* and *Asaia* were fed in combination the total concentration was also adjusted to 2- 4 × 10^7^ CFU mL^-1^. The microbial suspensions were fed to 3 day old sand flies via a Hemotek membrane through chick skin feeders. Control flies were fed on sterile 7% sucrose solution. Membrane feeding of bacteria and selection of only fully engorged insects was done to standardise the amount of bacteria ingested. Fully engorged female sand flies were allowed to rest for 96 h, prior to *Leishmania* infection using 10^6^ promastigotes per mL in heat-inactivated rabbit blood. Sand flies were dissected at 72 h after *Leishmania* infection and the numbers of promastigotes were counted using a haemocytometer.

To investigate the effect of dose on colonisation resistance towards *Leishmania* an *O. intermedium* culture was grown overnight in Luria-Bertani medium and diluted to 1 × 10^6^ and 1 × 10^7^CFU mL^-1^. The bacterial suspensions were prepared and fed to flies as described above. The effect of live bacteria on colonisation resistance was assessed by pre-feeding heat inactivated *O. intermedium* (80°C for 15 minutes with a 10^7^ CFU mL^-1^ culture) and monitoring *Leishmania* development within the sand fly gut in comparison to a live *O. intermedium* culture at the same concentration.

### Incubation of *L. mexicana*with yeast and bacteria *in vitro*

*Pseudozyma sp*, *Ochrobactrum intermedium* and *Asaia* sp. were inoculated in liquid LB media and incubated overnight. Cells without media were obtained by centrifuging the cultures at 6,500 g for 5 minutes and washing cells twice with sterile PBS. Culture media free of cells was obtained by filtering cultures with 0.22 μM filters prior to incubation with *Leishmania. L. mexicana* promastigotes were centrifuged at 2,000 g for 5 minutes, washed twice with sterile 1 × PBS and re-suspended to 6 × 10^6^ promastigotes mL^-1^. A 1:1 mixture of *L. mexicana* promastigotes (3 × 10^6^ final concentration) were incubated either with cells (10^7^ CFU mL^-1^ final concentration) or previously used culture media at 26°C for 24 h. The number of surviving promastigotes was recorded using a haemocytometer.

### *L. mexicana*incubation with *S. marcescens in vitro*

The *L. mexicana* promastigotes were centrifuged at 1,500 g for 10 minutes, washed twice with sterile PBS and re-suspended in PBS. *Serratia marcescens* was inoculated in liquid Luria-Bertani media and grown overnight as described above. After a centrifugation at 6,500 g for 5 minutes, culture media was discarded; bacterial cells were washed twice in PBS. 1 mL of re-suspended bacterial cells (final concentration of 10^7^ CFUmL^-1^) was incubated overnight with 1 mL of *Leishmania* promastigotes (final concentration 3 × 10^6^ parasites mL^-1^) at 26°C. Incubations were repeated three times in triplicate and the number of *Leishmania* promastigotes was recorded using a haemocytometer at 24 hours after incubation. For incubations with culture supernatant the *S. marcescens* was inoculated in liquid Luria-Bertani media and grown overnight at 37°C (200 rpm). Spent medium from *Serratia* culture was produced by filtering (0.22 μm filter) a bacterial culture (5.7 × 10^7^ CFU mL^-1^). The spent media and parasites were incubated together and assessed as described above.

### *Leishmania*DNA quantification by qPCR

*Leishmania* DNA quantification in adult females of *Lu. longipalpis* was performed as previously described [30]. Briefly, 50 ng of sand fly DNA from 12 pools of 5 insects was used in a real time PCR reaction using primers that amplify a 120-bp fragment of the *Leishmania* minicircle kDNA. A standard curve was constructed using DNA from *L. mexicana* (strain MNYC/BZ/62/M379) obtained from promastigote cultures (3 × 10^7^ mL^-1^) in a 10 fold dilution series ranging from 10 ng to 100 fg.

### Bacterial quantification in laboratory reared sand flies

Detection of bacterial DNA by quantitative PCR was performed as previously described [23]. Briefly, 6 pools of 5 newly emerged female *Lu. longipalpis* (approximately 1 h after emergence), 3 day old flies sugar fed with sterile 70% sucrose solution changed daily and blood fed flies at 1, 24 and 72 h after blood feed were surface-sterilised in 70% ethanol for 1 min and rinsed in 1 × PBS for 1 min. Total DNA was extracted, diluted to a final concentration of 50 ng of DNA μl^-1^ and added to the quantitative PCR mix. Bacterial copy numbers were calculated using *Asaia* DNA as standard and universal bacterial primers [31].

### *Leishmania*and *Serratia*co-infection of sand flies

Female sand flies were infected with *L. mexicana* 3 days after emergence as described above. Infected sand flies were transferred to a new cage and maintained with autoclaved 70% w/v sucrose solution on cotton wool for 4 days. *Leishmania*-infected sand flies were then orally challenged with *Serratia,* re-suspended to give a concentration of 5.7 × 10^7^ cfu mL^-1^ in autoclaved 20% w/v sucrose solution and fed to *Leishmania*-infected sand flies daily via cotton wool. Cotton wool moistened with *Serratia* solution was changed daily and sand fly survival was monitored for 6 days after bacterial challenge and compared with uninfected bloodfed sand flies that were also challenged with *Serratia*. Additional control groups consisted of *Leishmania*-infected and bloodfed sand flies fed with sterile 20% w/v sucrose solution via cotton wool. Sand fly *Leishmania* infections were estimated by dissecting infected sand fly guts at 72 h after starting *Serratia* feeding. Bacterial population size was also estimated in the gut by dissecting insects 72 h post-infection, four pools of 3 midguts were homogenised in 50 μl of PBS per pool and serial dilutions were inoculated onto LB agar plates and incubated at 26°C for 24 h.

### Statistical analysis

Survival analyses were performed using the Kaplan-Meier Log Rank χ2 test. Multiple comparisons were carried out with Kruskal-Wallis and pair-wise comparisons using the Mann-Whitney *U* test and Fishers Exact test. Results are expressed as the group mean ± SEM. Significance was considered when *p* < 0.05. All data were analysed with SPSS Data Editor Software (version 17.0, SPSS Inc).

## Results and discussion

### Effect of commensal microorganisms on *Leishmania*infection of *Lutzomyia longipalpis*

A wide range of microbial phylotypes have been found associated with adult phlebotomine sand flies collected in the field [4–7, 32] and the percentage of field caught females found to contain a *Leishmania* population in their gut were usually below 1% [33–35]. Thus *Leishmania* is only one of many microorganisms vying to occupy the sand fly gut. In the first part of the study we assessed the effect of the interaction between gut microorganisms with *Leishmania* on subsequent successful colonisation. We used a yeast, *Pseudozyma sp.* and a bacterium *Asaia sp.* isolated from the gut of female sand flies collected in a region endemic for visceral leishmaniasis and also *O. intermedium*, present in our sand fly colony (and other colonies [8]). The α-proteobacterium *Asaia* used in these experiments was the first of this genus to be isolated from a New World sand fly; in this case a female *Lu. longipalpis* from a chicken house in Teresina (Piauí-Brazil). There is one record of *Asaia* being present in a female *Phlebotomus sergentii* [36]. *Asaia* were previously isolated from anopheline mosquitoes and are highly prevalent and abundant in their midgut microbiota [37, 38]. It is interesting that both *Asaia* and the yeast-like fungus *Pseudozyma* are associated with plants and have osmophilic properties [39, 40] and may have been acquired by the female during plant feeding for sugar-rich food.

**Figure 1:**
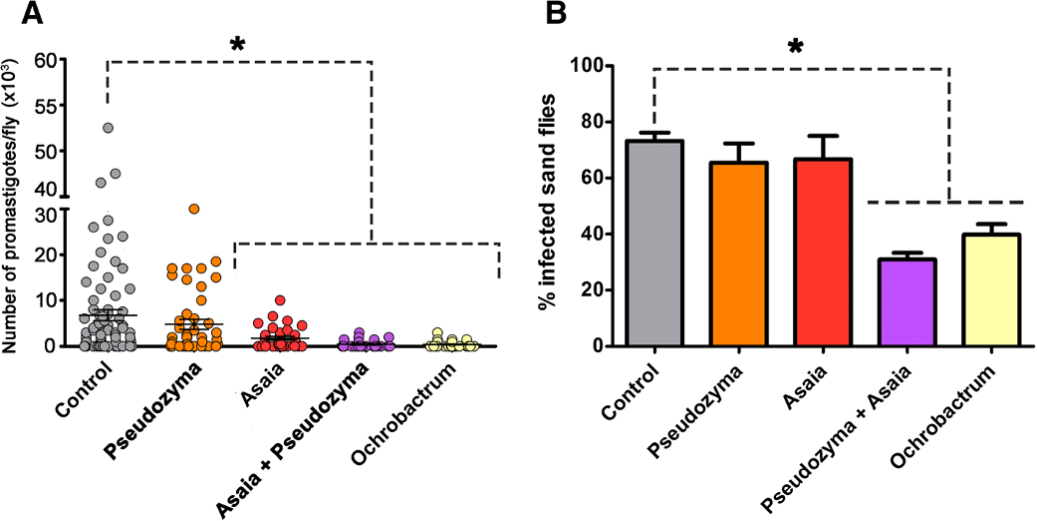
**Impact of pre-feeding bacteria and yeast on the subsequent** ***Leishmania*** **population within the gut of female** ***Lu. longipalpis*** **. (A)** *Leishmania* promastigote population estimated within the midgut of *Lu. longipalpis* after feeding with *Pseudozyma* sp; *Asaia* sp. or *O. intermedium;* 2 - 4 × 10^7^ CFU mL^-1^. Circles represent individual parasite counts in individual sand fly midguts from 3 independent experiments for each microbial species. *Kruskal-Wallis: *P* ≤ 0.0001. Mann-Whitney U test: *P* ≤ 0.007. **(B)** Percentage of female flies infected with *Leishmania*. Control group were fed on 7% w/v sucrose before being fed with *Leishmania*. *Fisher’s Exact Test P ≤ 0.0001.

To investigate the microbe-*Leishmania* interaction *in vivo*, female *Lu. longipalpis* were given a yeast or a bacterial feed 4 days prior to being membrane-fed with *Leishmania mexicana* in a bloodmeal. The impact upon the *Leishmania* population was evaluated by estimating the number of *Leishmania* promastigotes inside the sand fly gut 72 h after the bloodmeal and the number of sand flies infected were compared to control flies (Figure 1). Prior colonisation by *Asaia* and *O. intermedium* significantly reduced the size of the *Leishmania* populations within the sand flies (Figure 1A) and with *O. intermedium*, the sand fly infection rates were also reduced (Figure 1B). We also investigated the effect of pre-feeding a combination of two strains of microorganisms, *Pseudozyma* with *Asaia,* and there was a significant reduction both in the size of the *Leishmania* populations and in the number of sand flies containing a *Leishmania* infection after pre-feeding with the mixed culture (Figure 1A and 1B). This result is consistent with predictions of community ecology theory applied to insect gut microbiota [41] that suggest increasing species diversity within the sand fly gut, by feeding two rather than one species, would lead to a community more resistant to invasion by the *Leishmania*. It is also possible that the other gut bacteria already present, albeit at low levels (Day 3 onwards: Additional file 1: Figure S2), may have contributed to the colonisation resistance seen towards the *Leishmania.*

**Figure 2:**
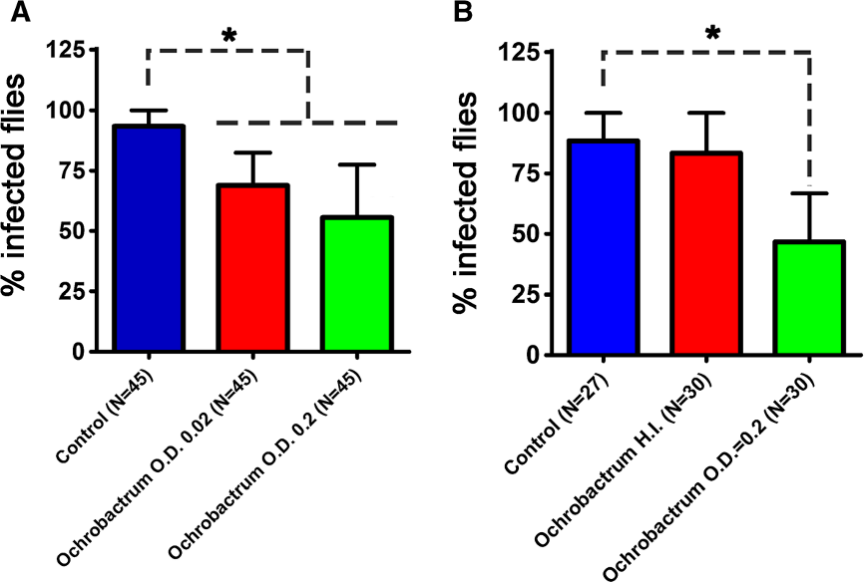
**Effect of** ***O. intermedium*** **concentration and heat inactivation on the percentage of female** ***Lu. Longipalpis*** **containing a** ***Leishmania*** **infection. (A)** Two concentrations of *O. intermedium* of 10^6^ (OD 0.02) and 10^7^ CFU mL^-1^ (OD 0.2) were used to feed groups of sand flies prior to *Leishmania* infection. Control group were fed on 7% sucrose only before infections. Three independent experiments were carried out. Fisher’s Exact Test:* *P* ≤ 0.0001. **(B)** Effect of feeding heat inactivated (HI) *O. intermedium* (10^7^ CFU mL^-1^) on subsequent *Leishmania* percentage infection of females. Infection rates from individual sand fly midguts from 3 independent experiments. Fisher’s Exact Test: *P ≤ 0.0001.

The effects were further studied by comparing two *O. intermedium* concentrations of 10^6^ and 10^7^ cfu mL^-1^ fed to female *Lu. longipalpis* 4 days prior to being fed with *L. mexicana*. The results showed that there was no concentration dependent effect as the *Leishmania* promastigote population was not reduced when flies were membrane-fed with *O. intermedium* at 10^7^ CFU mL^-1^, in comparison with flies fed the lower concentration of 10^6^ CFU mL^-1^ (Figure 2A). A sand fly is estimated to ingest less than 0.8 μl of fluid in a meal [19], this suggests that an initial dose of < 800 bacteria was sufficient to reduce the subsequent *Leishmania* population in sand flies. We also showed that the *Ochrobactrum* needed to be alive to exert these effects, as the *Leishmania* infection rates observed in sand flies fed with heat-inactivated bacteria at 10^7^ CFU mL^-1^ were not significantly different from the controls (Figure 2B). This result together with the observation that only bacterial or yeast cells, and not supernatant, showed growth limiting effects towards *Leishmania in vitro* (Additional file 2: Figure S1A & B) suggest that microbial interference in *Leishmania* development is likely to occur only when the live cells are present.

The sand flies used in the colonisation study were reared in an aseptic environment with minimal microbial contamination but were not “germ free”. The aseptically prepared adults were assessed using QPCR for the bacterial 16S rDNA gene and the results indicated a low level of bacteria associated with these insects that did not change significantly after bloodfeeding (Additional file 1: Figure S2). Newly emerged adults were associated with significantly larger amounts of bacterial DNA that were not evident in 3 day old adults. We suggest that the bacterial DNA was associated with the meconium [42] that would have contained bacteria (dead or alive) as remnants of the larval gut. The meconium is voided from the adult gut within hours of the adult emerging from the pupae. Therefore, the sand flies may have contained gut bacteria carried over from the larval stages after pupation or from environmental contamination. Antibiotic treatment to eliminate the gut bacteria was not used as relevant antibiotics would potentially also inhibit *Leishmania* development. Germ free insects were not used as the immune response of these insects may differ markedly from a conventionally reared insect [43] and the immune response may form a vital function as part of the interaction between sand fly host, *Leishmania* and bacteria or yeast [23, 44].

The majority of the studies describing the effects of gut microbiota on parasite prevalence and development within medically important insect vectors have been done with mosquitoes [45], tsetse flies [13] and triatomine bugs [12]. *Plasmodium* is only found within the mosquito midgut for a limited period of time in comparison to *Leishmania* in the gut of their insect vector. However, a positive correlation was observed between the presence of a midgut microbiota and inhibition of *Plasmodium* development within the mosquito [43]. This inhibition was variously attributed to direct competition [43], active production of ROS by indigenous mosquito microbiota [46] or a consequence of activation of the basal immunity generated by mosquito gut bacteria [47]. Although very dominant in anopheline microbiota, there is no evidence that the symbiotic *Asaia* induce colonisation resistance in mosquitoes [38].

Our previous studies indicate that immune activation of the sand fly can lead to loss of *Leishmania* infection [23] and that although *Leishmania* do not appear to activate a ROS response, bacteria in the gut do cause ROS activation [44]. Direct production of antimicrobial compounds by bacteria, as found in other insects [48] cannot be discounted. The mechanisms of colonisation resistance in sand flies are therefore likely to be multifaceted; including direct bacterial-mediated lysis, competition for binding sites and nutrients or indirect via immune priming of the sand fly host. An intriguing parallel situation is demonstrated in plant systems with the epiphytic *Pseudozyma* and plant pathogen *Botrytis cinerea* [49]. *Pseudozyma aphidis* secreted extracellular metabolites not only inhibit the pathogen but also primes the plant immune system to invoke a local and systemic immune response towards the pathogen.

### *Role of Leishmania*in protecting *Lutzomyia longipalpis*from an insect pathogen

**Figure 3:**
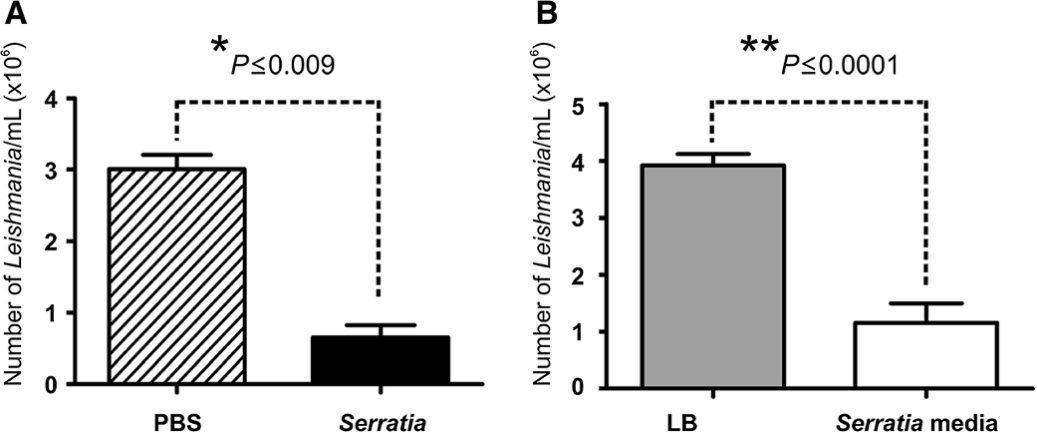
**Effect of** ***S. marcescens*** **on growth of** ***L.*** **mexicana** ***in vitro*** **. (A)** *In vitro* 24 h incubation of *Serratia* bacterial cells (10^7^ CFU mL^-1^) or **(B)** filtered spent medium from *Serratia* culture with *L. mexicana* (3 × 10^6^ promastigotes mL^-1^). Results are based on triplicate samples repeated three times and bar charts represent mean ± SEM. *P ≤ 0.009. **P ≤ 0.0001 (Mann-Whitney U test).

The final part of the investigation addressed the hypothesis that *Leishmania* are beneficial to the sand fly host. There are many microbial species encountered by the sand fly vector that are potentially pathogenic to the insect. We investigated circumstances in which the *Leishmania* may prevent the development of a sand fly pathogen. The colonisation experiments were repeated but the feeding regime was reversed; we fed bacteria to sand flies already colonised with *Leishmania*. We used the insect bacterial pathogen, *Serratia marcescens* as it is known to be associated with wild *Lu. longipalpis* and is also lethal to *Leishmania in vitro* [7, 25]. Preliminary *in vitro* experiments confirmed that both the cells of *S. marcescens* and the spent culture media incubated with our strain of *L. mexicana* led to suppression of parasite growth (Figure 3).

**Figure 4:**
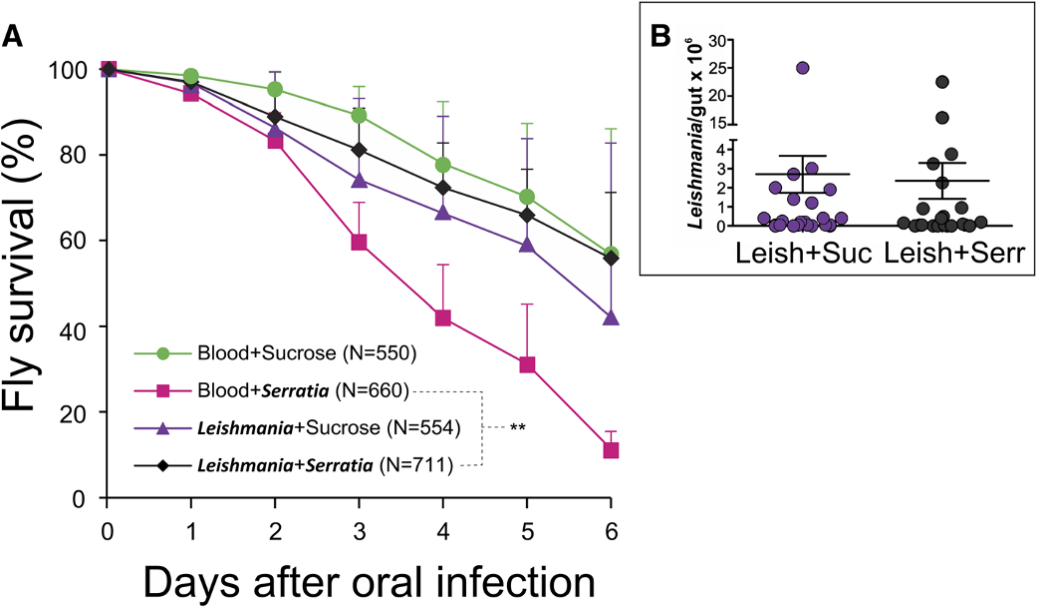
**Effect of** ***Leishmania*** **infection on sand fly survival after oral challenge with** ***Serratia marcescens*** **. (A)** Survival of female *Lu. longipalpis* containing *Leishmania* after oral challenge with *Serratia marcescens* in sucrose (diamond) in comparison with insects fed with a bloodmeal containing *Serratia* (square), *Leishmania* (triangle), or blood followed by sucrose (circle). **P ≤ 0.0001; Chi-square 96.987 (Log Rank-Mantel Cox). **(B)** Scatter plot showing *Leishmania* promastigote population, at day 3, within individual sand fly midguts after subsequent feeding with 20% w/v sucrose or a *Serratia marcescens* suspension (5.7 × 10^7^ CFU mL^-1^) via cotton wool.

Flies that were blood fed and then subjected to a daily feed of *Serratia* in a sugar meal (Figure 4A) succumbed to the bacterial infection and only 11% survived after 6 days. In contrast, sand fly survival was significantly higher (56% after 6 days; Figure 4A) when fed a blood meal containing *L. mexicana* amastigotes and then subjected to a daily feed of *Serratia* in a sugar meal. The presence of *Leishmania* in the gut enhanced the survival of *Serratia*-challenged sand flies in comparison with those not infected with *Leishmania*. Remarkably, the population of *Leishmania* in these sand flies was not significantly different to those in a further set of control flies that were not fed *Serratia* (Figure 4B, Additional file 3: Figure S3). Survival of sand flies infected with *Leishmania* but not challenged with *Serratia* was no different to that of control sand flies (Figure 4A).

When an insect vector containing a medically important parasite is exposed to a pathogen of the vector it threatens the successful transmission of the parasite. In our experiments when the *Leishmania* infected sand flies were subsequently fed with *Serratia,* the *Leishmania* population was similar to that of the control insects. This suggests that the association between *Leishmania* and its insect vector promote both survival of the insect and the flagellate population. Additionally there was no difference in the survival of flies with *Leishmania* infection with or without the *Serratia* challenge during the period of the experiment.

**Figure 5:**
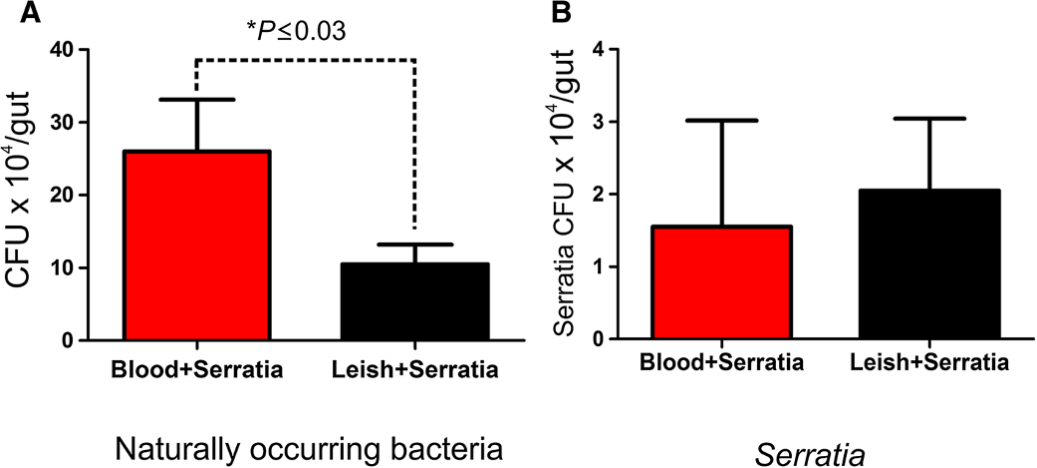
**Estimate of naturally-occurring sand fly gut bacteria. (A)** and *Serratia marcescens* **(B)** in *Leishmania* infected flies after oral challenge with *Serratia.* Estimated as CFUs present in individual *Lu. longipalpis* midguts either uninfected (Blood + *Serratia*) or infected with *L. mexicana* (Leish + *Serratia*) at 3 days after daily oral challenge with *Serratia marcescens* via cotton wool (5.7 × 10^7^ *Serratia* CFUmL^-1^, resuspended with sterile 20% w/v sucrose solution). Asterisk represents statistical difference using Mann-Whitney U test (*P* ≤ 0.03) of at least two independent experiments.

There was a significant reduction in the population of naturally-occurring sand fly gut bacteria sampled at 3 days (Figure 5A) in *Leishmania* infected sand flies that had been exposed to a daily *Serratia* oral challenge. However, there were no differences in *Serratia* populations in surviving flies infected with *Leishmania* in comparison with the flies without *Leishmania* (Figure 5B). The decrease in gut microbiota is consistent with the idea that colonisation resistance was generated by *Leishmania* towards indigenous bacteria within the sand fly. It should be noted that sampling of blood fed sand flies without *Leishmania* may have resulted in a lower than expected population of *Serratia* since flies with higher *Serratia* doses probably died before the time of sampling.

These experiments highlighted one circumstance in which the association between *Leishmania* and sand flies may be mutually beneficial. Increased protection from insect pathogens will extend the lifespan of sand flies. But does the potential benefit of the *Leishmania* help to offset the known fitness costs [18] of a *Leishmania* population in the sand fly gut? This is a significant question since even a few days life extension in a disease endemic area may greatly promote *Leishmania* transmission and contribute to the successful spread of the human disease. An increased disease resistance conferred on the insect by *Leishmania* could be an important evolutionary pressure for the maintenance of the *Leishmania*-vector association. Sand flies more resistant to *Leishmania* infection may be more exposed to enteric bacterial entomopathogens. In this case maintenance of a small fraction of *Leishmania*-susceptible flies in the vector species may ensure insect survival within a population that is succumbing to an entomopathogen.

A further implication of the protective effect of *Leishmania* is that implementing a biological control campaign against insect vectors using insect pathogens may have unwarranted effects. Sand flies not carrying *Leishmania* may succumb more rapidly to the biological control agent and this would lead to the development of a wild sand fly population containing an increased proportion of the surviving flies carrying the human disease agent. Any new forms of control aimed at insect vectors of human disease need to consider the total macro- and micro- ecology of the relationship between the insect and the human parasite.

## Conclusions

The colonisation experiments show that pre-feeding yeast or bacteria to the sand fly can prevent the establishment of *Leishmania* within the sand fly vector. There are many factors governing the prevalence of leishmaniasis within endemic areas [50], but the possibility that the species composition and abundance of microbiota in the female sand fly may influence *Leishmania* transmission should be considered in modelling the spread of the disease.

Our study also showed that sand flies infected with *Leishmania* were able to survive an attack by a bacterial pathogen that would have killed the insect and we concluded that *Leishmania* may benefit its insect host whilst increasing the potential to establish itself in the sand fly vector. We suggest that this is evidence for a mutually beneficial interaction between the vector fly and *Leishmania.* Furthermore, this may be an example of a subtle and intermittent interaction existing in the natural habitat that provides an evolutionary pressure for the maintenance of the sand fly - *Leishmania* association.

## Electronic supplementary material

Additional file 1: Figure S2: Bacterial DNA copy number in newly emerged and aseptically reared sugar-fed (3 day old) and blood fed (BF) female *Lu. longipalpis*. Bars represent bacterial copy number of 6 pools of 5 ethanol-sterilised and PBS-washed sand flies. Asterisk represents statistical difference (Mann-Whitney U test, *P* ≤ 0.05). (TIFF 8 MB)

Additional file 2: Figure S1: Effect of *In vitro* incubation of yeast and bacterial cells and media on the growth of *L. mexicana*. **(A)** Number of *L. mexicana* promastigotes after *in vitro* incubation with *Pseudozyma* sp*.*, *Ochrobactrum intermedium* and *Asaia* sp. (10^7^ CFU mL^-1^) and **(B)** microbiological media for 24 h at 26°C. PBS and LB media were used as a control. Experiments were done in triplicate and repeated three times. Mann Whitney U test: a- *P < 0.044*; b- *P < 0.001*; c- *P < 0.0045.* (ZIP 75 KB)

Additional file 3: Figure S3: *Leishmania* minicircle kDNA copy number determined by qPCR. Presence in 12 pools of 5 sand flies infected with *L. mexicana* and subsequently fed with 20% w/v sucrose (Leish + sucrose) or a *Serratia* suspension (Leish + *Serratia* - 5.7 × 10^7^ CFUmL^-1^ prepared in autoclaved 20% w/v sucrose ) via cotton wool for 6 days. Bar charts represent mean ± SEM of three independent experiments (*P >* 0.05). (TIFF 3 MB)

## References

[1] Killick-Kendrick R (1999). The biology and control of phlebotomine sand flies. Clin Dermatol.

[2] Ready PD (2013). Biology of phlebotomine sand flies as vectors of disease agents. Annu Rev Entomol.

[3] Bates PA (2008). *Leishmania* sand fly interaction: progress and challenges. Curr Opin Microbiol.

[4] Dillon RJ, el Kordy E, Shehata M, Lane RP (1996). The prevalence of a microbiota in the digestive tract of *Phlebotomus papatasi*. Ann Trop Med Parasitol.

[5] Sant'anna MR, Darby AC, Brazil RP, Montoya-Lerma J, Dillon VM, Bates PA, Dillon RJ (2012). Investigation of the bacterial communities associated with females of *Lutzomyia* sand fly species from South America. PLoS One.

[6] Hillesland H, Read A, Subhadra B, Hurwitz I, McKelvey R, Ghosh K, Das P, Durvasula R (2008). Identification of aerobic gut bacteria from the kala azar vector, *Phlebotomus argentipes*: a platform for potential paratransgenic manipulation of sand flies. Am J Trop Med Hyg.

[7] McCarthy CB, Diambra LA, Rivera Pomar RV (2011). Metagenomic analysis of taxa associated with *Lutzomyia longipalpis*, vector of visceral leishmaniasis, using an unbiased high-throughput approach. PLoS Negl Trop Dis.

[8] Volf P, Kiewegova A, Nemec A (2002). Bacterial colonisation in the gut of *Phlebotomus duboscqi* (Diptera : Psychodidae): transtadial passage and the role of female diet. Folia Parasitol.

[9] Lawley TD, Walker AW (2013). Intestinal colonization resistance. Immunology.

[10] Dillon RJ, Dillon VM (2004). The gut bacteria of insects: nonpathogenic interactions. Annu Rev Entomol.

[11] Azambuja P, Garcia ES, Ratcliffe NA (2005). Gut microbiota and parasite transmission by insect vectors. Trends Parasitol.

[12] Castro DP, Moraes CS, Gonzalez MS, Ratcliffe NA, Azambuja P, Garcia ES (2012). *Trypanosoma cruzi* immune response modulation decreases microbiota in *Rhodnius prolixus* gut and Is crucial for parasite survival and development. PLoS One.

[13] Wang J, Weiss BL, Aksoy S (2013). Tsetse fly microbiota: form and function. Frontiers in cellular and infection microbiology.

[14] Cirimotich CM, Ramirez JL, Dimopoulos G (2011). Native microbiota shape insect vector competence for human pathogens. Cell Host Microbe.

[15] Schlein Y, Polacheck I, Yuval B (1985). Mycoses, bacterial infections and antibacterial activity in sandifies (Psychodidae) and their possible role in the transmission of leishmaniasis. Parasitology.

[16] Casanova C, Natal D, Santos FAM (2009). Survival, population size, and gonotrophic cycle duration of *Nyssomyia neivai* (Diptera: Psychodidae) at an endemic area of American cutaneous leishmaniasis in southeastern Brazil. J Med Entomol.

[17] Rogers ME, Ilg T, Nikolaev AV, Ferguson MAJ, Bates PA (2004). Transmission of cutaneous leishmaniasis by sand flies is enhanced by regurgitation of fPPG. Nature.

[18] Rogers ME, Bates PA (2007). *Leishmania* manipulation of sand fly feeding behavior results in enhanced transmission. Plos Pathogens.

[19] Dillon RJ, Lane RP (1993). Influence of *Leishmania* infection on blood-meal digestion in the sandflies *Phlebotomus papatasi* and *Phlebotomus langeroni*. Parasitol Res.

[20] Sant'Anna M, Diaz-Albiter H, Mubaraki M, Dillon R, Bates P (2009). Inhibition of trypsin expression in *Lutzomyia longipalpis* using RNAi enhances the survival of *Leishmania*. Parasit Vectors.

[21] Telleria EL, Pitaluga AN, Ortigao-Farias JR, de Araujo AP, Ramalho-Ortigao JM, Traub-Cseko YM (2007). Constitutive and blood meal-induced trypsin genes in *Lutzomyia longipalpis*. Arch Insect Biochem Physiol.

[22] Rogers ME, Hajmova M, Joshi MB, Sadlova J, Dwyer DM, Volf P, Bates PA (2008). *Leishmania* chitinase facilitates colonization of sand fly vectors and enhances transmission to mice. Cell Microbiol.

[23] Telleria EL, Sant'Anna MR, Ortigao-Farias JR, Pitaluga AN, Dillon VM, Bates PA, Traub-Cseko YM, Dillon RJ (2012). Caspar-like gene depletion reduces *Leishmania* infection in sand fly host *Lutzomyia longipalpis*. J Biol Chem.

[24] Harhay MO, Olliaro PL, Costa DL, Costa CH (2011). Urban parasitology: visceral leishmaniasis in Brazil. Trends Parasitol.

[25] Moraes CS, Seabra SH, Castro DP, Brazil RP, de Souza W, Garcia ES, Azambuja P (2008). *Leishmania (Leishmania) chagasi* interactions with *Serratia marcescens*: ultrastructural studies, lysis and carbohydrate effects. Exp Parasitol.

[26] Modi GB, Tesh RB (1983). A simple technique for mass rearing *Lutzomyia longipalpis* and *Phlebotomus papatasi* (Diptera: Psychodidae) in the laboratory. J Med Entomol.

[27] Webster G, Parkes RJ, Cragg BA, Newberry CJ, Weightman AJ, Fry JC (2006). Prokaryotic community composition and biogeochemical processes in deep subseafloor sediments from the Peru Margin. FEMS Microbiol Ecol.

[28] Kurtzman C, Robnett C (1998). Identification and phylogeny of ascomycetous yeasts from analysis of nuclear large subunit (26S) ribosomal DNA partial sequences. Antonie Van Leeuwenhoek.

[29] Sant'Anna MRV, Alexander B, Bates PA, Dillon RJ (2008). Gene silencing in phlebotomine sand flies: Xanthine dehydrogenase knock down by dsRNA micro-injections. Insect Biochem Mol Biol.

[30] Nicolas LPE, Lang T, Milon G (2002). Real-time PCR for detection and quantitation of *Leishmania* in mouse tissues. J Clin Microbiol.

[31] Nadkarni MA, Martin FE, Jacques NA, Hunter N (2002). Determination of bacterial load by real-time PCR using a broad-range (universal) probe and primers set. Microbiol.

[32] Gouveia C, Asensi MD, Zahner V, Rangel EF, Oliveira SM (2008). Study on the bacterial midgut microbiota associated to different Brazilian populations of *Lutzomyia longipalpis* (Lutz & Neiva) (Diptera: Psychodidae). Neotrop Entomol.

[33] Murray HW, Berman JD, Davies CR, Saravia NG (2005). Advances in leishmaniasis. Lancet.

[34] Rangel EF, Lainson R (2009). Proven and putative vectors of American cutaneous leishmaniasis in Brazil: aspects of their biology and vectorial competence. Mem Inst Oswaldo Cruz.

[35] Felipe IM AD, Kuppinger O, Santos MD, Rangel ME, Barbosa DS, Barral A, Werneck GL, Caldas Ade J (2011). *Leishmania* infection in humans, dogs and sandflies in a visceral leishmaniasis endemic area in Maranhão, Brazil. Mem Inst Oswaldo Cruz.

[36] Akhoundi M, Bakhtiari R, Guillard T, Baghaei A, Tolouei R, Sereno D, Toubas D, Depaquit J, Abyaneh MR (2012). Diversity of the bacterial and fungal microflora from the midgut and cuticle of Phlebotomine sand flies collected in North-Western Iran. PLoS One.

[37] Favia G, Ricci I, Damiani C, Raddadi N, Crotti E, Marzorati M, Rizzi A, Urso R, Brusetti L, Borin S, Mora D, Scuppa P, Pasqualini L, Clementi E, Genchi M, Corona S, Negri I, Grandi G, Alma A, Kramer L, Esposito F, Bandi C, Sacchi L, Daffonchio D (2007). Bacteria of the genus Asaia stably associate with *Anopheles stephensi*, an Asian malarial mosquito vector. Proc Natl Acad Sci U S A.

[38] Damiani C, Ricci I, Crotti E, Rossi P, Rizzi A, Scuppa P, Capone A, Ulissi U, Epis S, Genchi M, Sagnon N, Faye I, Kang A, Chouaia B, Whitehorn C, Moussa GW, Mandrioli M, Esposito F, Sacchi L, Bandi C, Daffonchio D, Favia G (2010). Mosquito-bacteria symbiosis: the case of *Anopheles gambiae* and *Asaia*. Microb Ecol.

[39] Crotti E, Damiani C, Pajoro M, Gonella E, Rizzi A, Ricci I, Negri I, Scuppa P, Rossi P, Ballarini P, Raddadi N, Marzorati M, Sacchi L, Clementi E, Genchi M, Mandrioli M, Bandi C, Favia G, Alma A, Daffonchio D (2009). *Asaia*, a versatile acetic acid bacterial symbiont, capable of cross-colonizing insects of phylogenetically distant genera and orders. Environ Microbiol.

[40] Wei YH, Lee FL, Hsu WH, Chen SR, Chen CC, Wen CY, Lin SJ, Chu WS, Yuan GF, Liou GY (2005). *Pseudozyma antarctica* in Taiwan: a description based on morphological, physiological and molecular characteristics. Bot Bull Acad Sin.

[41] Dillon RJ, Vennard CT, Buckling A, Charnley AK (2005). Diversity of locust gut bacteria protects against pathogen invasion. Ecol Lett.

[42] Moll RM, Romoser WS, Modrzakowski MC, Moncayo AC, Lerdthusnee K (2001). Meconial peritrophic membranes and the fate of midgut bacteria during mosquito (Diptera: Culicidae) metamorphosis. J Med Entomol.

[43] Dong Y, Manfredini F, Dimopoulos G (2009). Implication of the mosquito midgut microbiota in the defense against malaria parasites. PLoS Pathog.

[44] Diaz-Albiter H, Sant'Anna MR, Genta FA, Dillon RJ (2012). Reactive oxygen species-mediated immunity against *Leishmania mexicana* and *Serratia marcescens* in the sand phlebotomine fly *Lutzomyia longipalpis*. J Biol Chem.

[45] Gendrin M, Christophides GK, Manguin S (2013). The *Anopheles* Mosquito Microbiota and Their Impact on Pathogen Transmission. Anopheles mosquitoes - New insights into malaria vectors.

[46] Cirimotich CM, Dong YM, Clayton AM, Sandiford SL, Souza-Neto JA, Mulenga M, Dimopoulos G (2011). Natural Microbe-Mediated Refractoriness to *Plasmodium* Infection in *Anopheles gambiae*. Science.

[47] Cirimotich CM, Dong Y, Garver LS, Sim S, Dimopoulos G (2010). Mosquito immune defenses against *Plasmodium* infection. Dev Comp Immunol.

[48] Dillon RJ, Charnley AK (2002). Mutualism between the desert locust *Schistocerca gregaria* and its gut microbiota. Res Microbiol.

[49] Buxdorf K, Rahat I, Gafni A, Levy M (2013). The epiphytic fungus *Pseudozyma aphidis* induces jasmonic acid-and salicylic acid/nonexpressor of PR1-independent local and systemic resistance. Plant Physiol.

[50] Soares MR, Carvalho CC, Silva LA, Lima MS, Barral AM, Rebelo JM, Pereira SR (2010). Molecular analysis of natural infection of *Lutzomyia longipalpis* in an endemic area for visceral leishmaniasis in Brazil. Cad Saude Publ.

