# Supplementary figures and images for: Colonisation resistance in the sand fly gut: Leishmania protects Lutzomyia longipalpis from bacterial infection
Source: Parasit Vectors. 2014 Jul 23;7:329. doi: 10.1186/1756-3305-7-329 (PMC4112039; doi:10.1186/1756-3305-7-329)

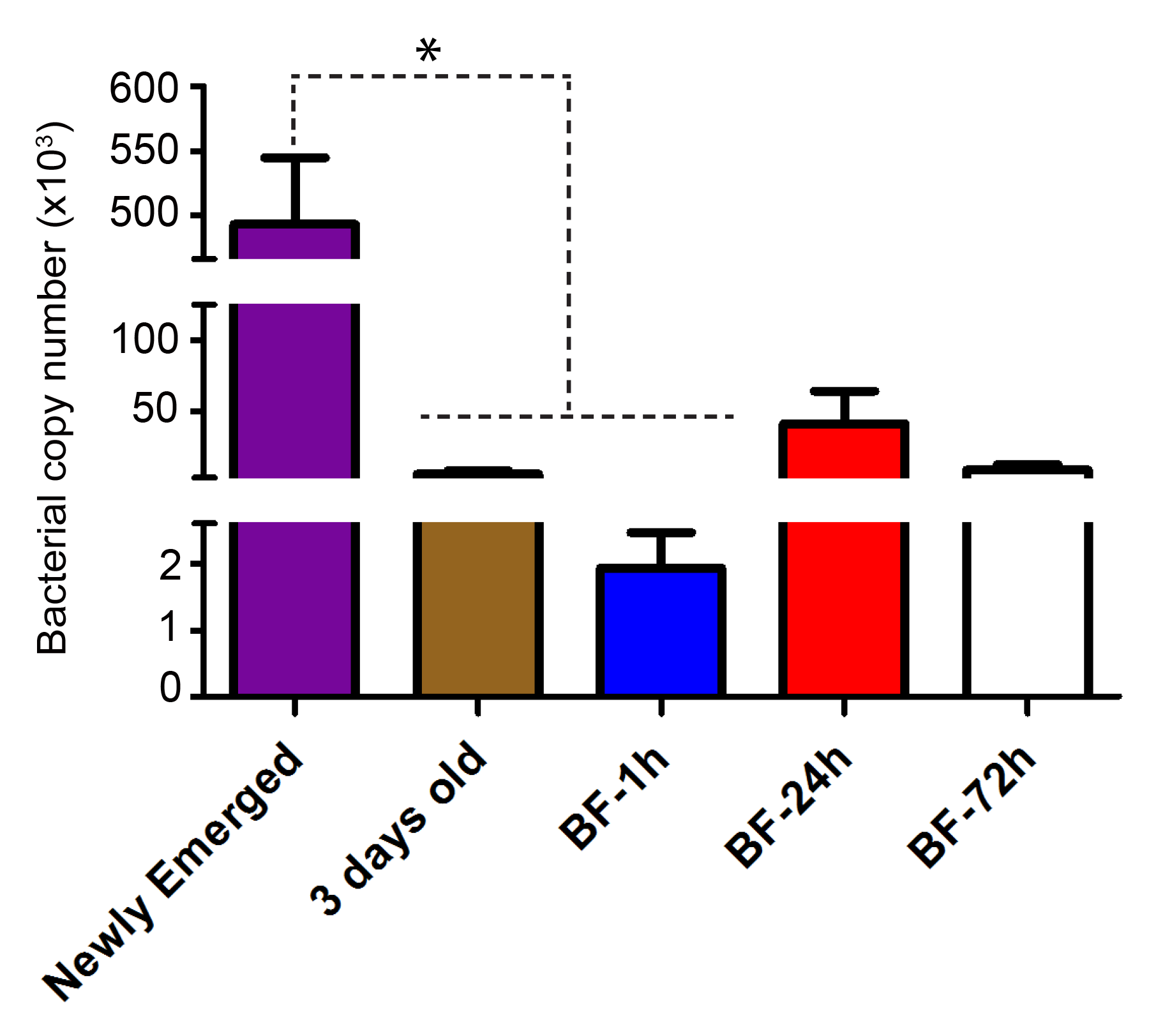

Supplement: Supplementary file 1 — Additional file 1: Figure S2: Bacterial DNA copy number in newly emerged and aseptically reared sugar-fed (3 day old) and blood fed (BF) female Lu. longipalpis. Bars represent bacterial copy number of 6 pools of 5 ethanol-sterilised and PBS-washed sand flies. Asterisk represents statistical difference (Mann-Whitney U test, P ≤ 0.05). (TIFF 8 MB) [file 13071_2014_1514_MOESM1_ESM.tiff]

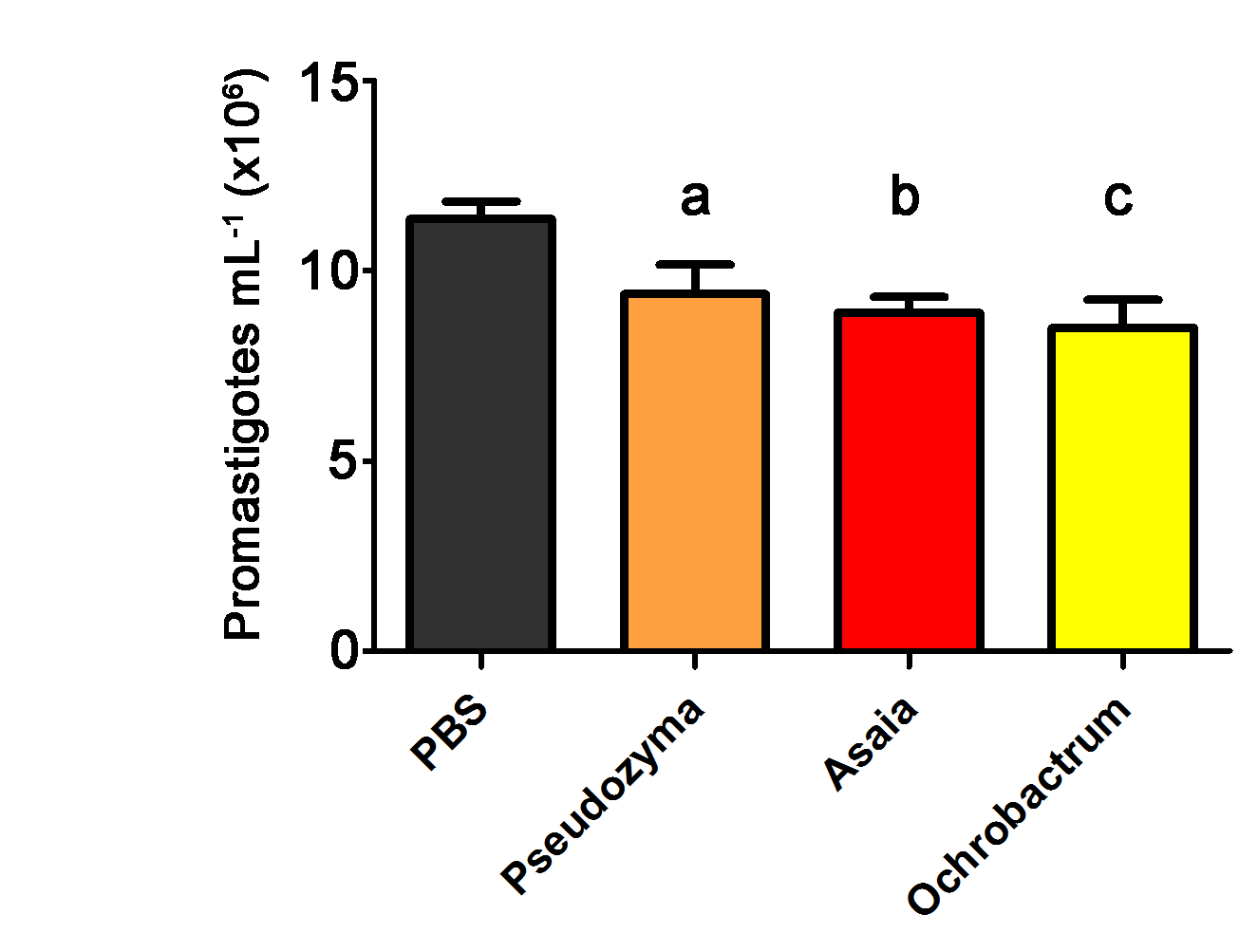

Supplement: Supplementary file 2 — Additional file 2: Figure S1: Effect of In vitro incubation of yeast and bacterial cells and media on the growth of L. mexicana. (A) Number of L. mexicana promastigotes after in vitro incubation with Pseudozyma sp., Ochrobactrum intermedium and Asaia sp. (107 CFU mL-1) and (B) microbiological media for 24 h at 26°C. PBS and LB media were used as a control. Experiments were done in triplicate and repeated three times. Mann Whitney U test: a- P < 0.044; b- P < 0.001; c- P < 0.0045. (ZIP 75 KB) [file 13071_2014_1514_MOESM2_ESM.zip › Figure S1A.tiff]

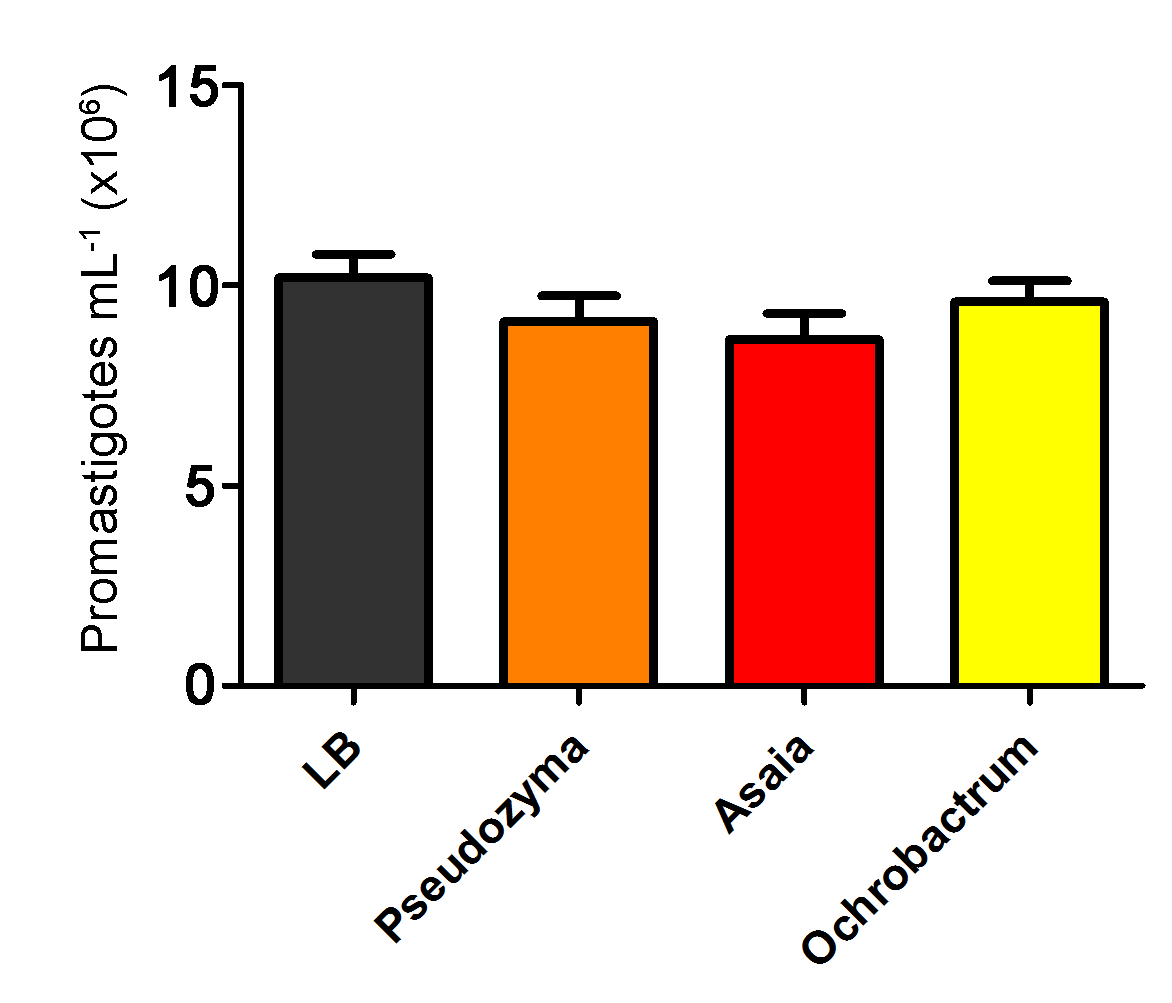

Supplement: Supplementary file 2 — Additional file 2: Figure S1: Effect of In vitro incubation of yeast and bacterial cells and media on the growth of L. mexicana. (A) Number of L. mexicana promastigotes after in vitro incubation with Pseudozyma sp., Ochrobactrum intermedium and Asaia sp. (107 CFU mL-1) and (B) microbiological media for 24 h at 26°C. PBS and LB media were used as a control. Experiments were done in triplicate and repeated three times. Mann Whitney U test: a- P < 0.044; b- P < 0.001; c- P < 0.0045. (ZIP 75 KB) [file 13071_2014_1514_MOESM2_ESM.zip › Figure S1B.tiff]

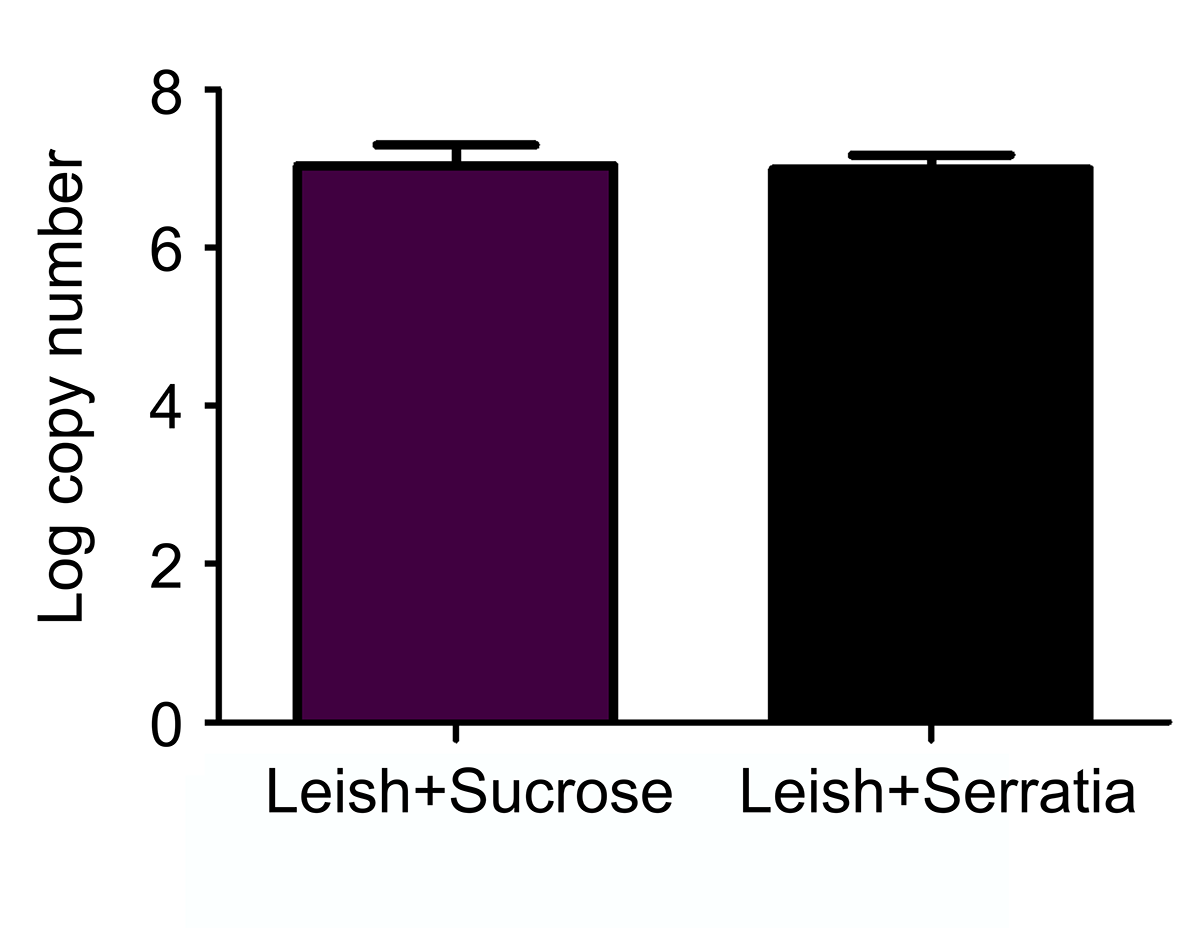

Supplement: Supplementary file 3 — Additional file 3: Figure S3: Leishmania minicircle kDNA copy number determined by qPCR. Presence in 12 pools of 5 sand flies infected with L. mexicana and subsequently fed with 20% w/v sucrose (Leish + sucrose) or a Serratia suspension (Leish + Serratia - 5.7 × 10^7^ CFUmL-1 prepared in autoclaved 20% w/v sucrose ) via cotton wool for 6 days. Bar charts represent mean ± SEM of three independent experiments (P > 0.05). (TIFF 3 MB) [file 13071_2014_1514_MOESM3_ESM.tiff]
